# Clinical characteristics and follow-up analysis of 324 discharged COVID-19 patients in Shenzhen during the recovery period

**DOI:** 10.7150/ijms.50873

**Published:** 2021-01-01

**Authors:** Han-Qing Liu, Bo Yuan, Ya-Wen An, Kai-Jing Chen, Qi Hu, Xiao-Peng Hu, Jia Zhao, Yu Dong, Yong-Xin Chen, Wei-Xin Li, Chang-Qing Sun, Jian-Chun Wang, Cheng Wang, Shuo Song

**Affiliations:** 1 Central Laboratory, Shenzhen Samii Medical Center, Shenzhen 518118, China.; 2 Department of Neurology, Shenzhen Samii Medical Center, Shenzhen 518118, China.; 3 School of Biological Sciences, Nanyang Technological University, Singapore 637551.; 4 Chinese Academy of Agricultural Sciences Agricultural Genomes Institute at Shenzhen, Genome Analysis Laboratory of the Ministry of Agriculture, Shenzhen 518118, China.; 5 Department of Neurosurgery, Shenzhen Samii Medical Center, Shenzhen 518118, China.; 6 Department of Clinical Laboratory, Shenzhen Samii Medical Center, Shenzhen 518118, China.

**Keywords:** Covid-19, vaccine, epidemiology, antibody

## Abstract

**Objectives:** Research on recovering COVID-19 patients could be helpful for containing the pandemic and developing vaccines, but we still do not know much about the clinical features, recovery process, and antibody reactions during the recovery period.

**Methods:** We retrospectively analysed the epidemiological information, discharge summaries, and laboratory results of 324 patients.

**Results:** In all, 15 (8.62%) patients experienced chest distress/breath shortness, where 8 of the 15 were severely ill. This means severely ill patients need an extended amount of time to recover after discharge; next, 20 (11.49%) patients experienced anxiety and 21 (12.07%) had headache/insomnia and a small fraction of them complained of anosmia/ageusia, indicating that these patients need treatment for mental and psychological health issues. Regarding the re-positive patients, their CT and laboratory test results showed no obvious evidence of illness progress or infectivity but a high anti-SARS-CoV-2 antibody expression.

**Conclusion:** Recovered COVID-19 patients need psychological and physiological care and treatment, re-positivity can occur in any person, but juveniles, females, and patients with mild/moderate existing symptoms have higher rates of re-positivity, While there is no evidence that turning re-positive has an impact on their infectivity, but it still alerted us that we need differentiate them in the following managements.

## Introduction

Coronavirus disease 2019 (COVID-19), caused by the severe acute respiratory syndrome coronavirus-2 (SARS-CoV-2), has affected most countries in the world. Until 14 July 2020, more than 12 million people have been confirmed to be infected and 570,000+ have died 1. There are many clinical studies on COVID-19 patients, but very few studies have focused on discharged patients and their follow-up. The fact that an increasing number of people are getting infected and are also asymptomatic is concerning 2. Furthermore, discharged patients have been found to show re-positive results on nucleic acid testing 3,4. Some of the discharged patients showed positive re-test results and become asymptomatic carriers with viral shedding, which means they are potentially infectious 5, and thus further measures like quarantine and regular nucleic acid testing should be taken 6. There are 3.4 million recovered and discharged patients globally and a portion could be asymptomatic carriers, so it is reasonable and necessary to test them regularly and to evaluate their physiological, mental, and social health and treat them appropriately 7. Shenzhen is a megacity with a population of more than 10 million, and it was one of the earliest cities to implement the 14-day medical isolation observation policy. In this study, we have included all patients who underwent 14 days of medical isolation during the past 3 months; we included recovered and discharged patients, asymptomatic carriers, imported cases, and re-positive patients. We analysed their epidemiologic and clinical features and laboratory and radiology results to evaluate their physiological and mental changes and sequela of treatment and provide references for further helping and managing them.

## Methods

### Cohort information and data collection

As we described previously 8, from 21 February, all COVID-19 patients who reached discharge criteria 9 were requested to stay in a designated medical centre for an extra 14-day medical isolation observation. This study involved all the 324 patients in Shenzhen Samii Medical Center who were instructed to undergo isolation observation from 21 February to 21 May, and we collected their epidemiological survey information, discharge summaries, laboratory tests results, and feedback at a random follow-up after they went home. The cut-off date for data collection was 21 June, so the patients had stayed at home for at least 1 full month. We categorized the 324 patients in three groups: 20 asymptomatic carriers were in Group A, 243 patients with mild or moderate symptoms were in Group M, and 61 with severe/critical symptoms were in Group S.

According to the relevant regulations, all clinical and laboratory tests beside nucleic acid tests are voluntary, so the test data are present in fragments.

This study was approved by the Shenzhen Samii Medical Center Institutional Review Board, and written informed consent was obtained from all patients. We declare that these data do not contain any private information of the patients. All methods were performed in accordance with the relevant guidelines and regulations.

### Statistics

Variables are described using median, interquartile range (IQR), mean ± standard deviation (SD), or percentages. The Mann-Whitney *U* test was used to perform the significant difference analysis of the basic information. A two-tailed independent sample t-test was used to show the significant difference in antibodies detected between non-re-positives vs re-positives and Group M and Group S. The correlation plot was performed using the corplot package based on clinical indicator at different stages, and the trend line of main immune parameters were performed using mean values in stages. The kernel density plot was performed using the ggplot2 package based on age-distribution data; the Mann-Whitney *U* test was conducted with ggplot2 and the two-tailed independent sample t-test with the ggpubr package of R software (v3.6).

Detailed methods are provided in supplementary methods.

## Results

### Epidemiological and clinical features of discharged COVID-19 patients

**Figure 1:**
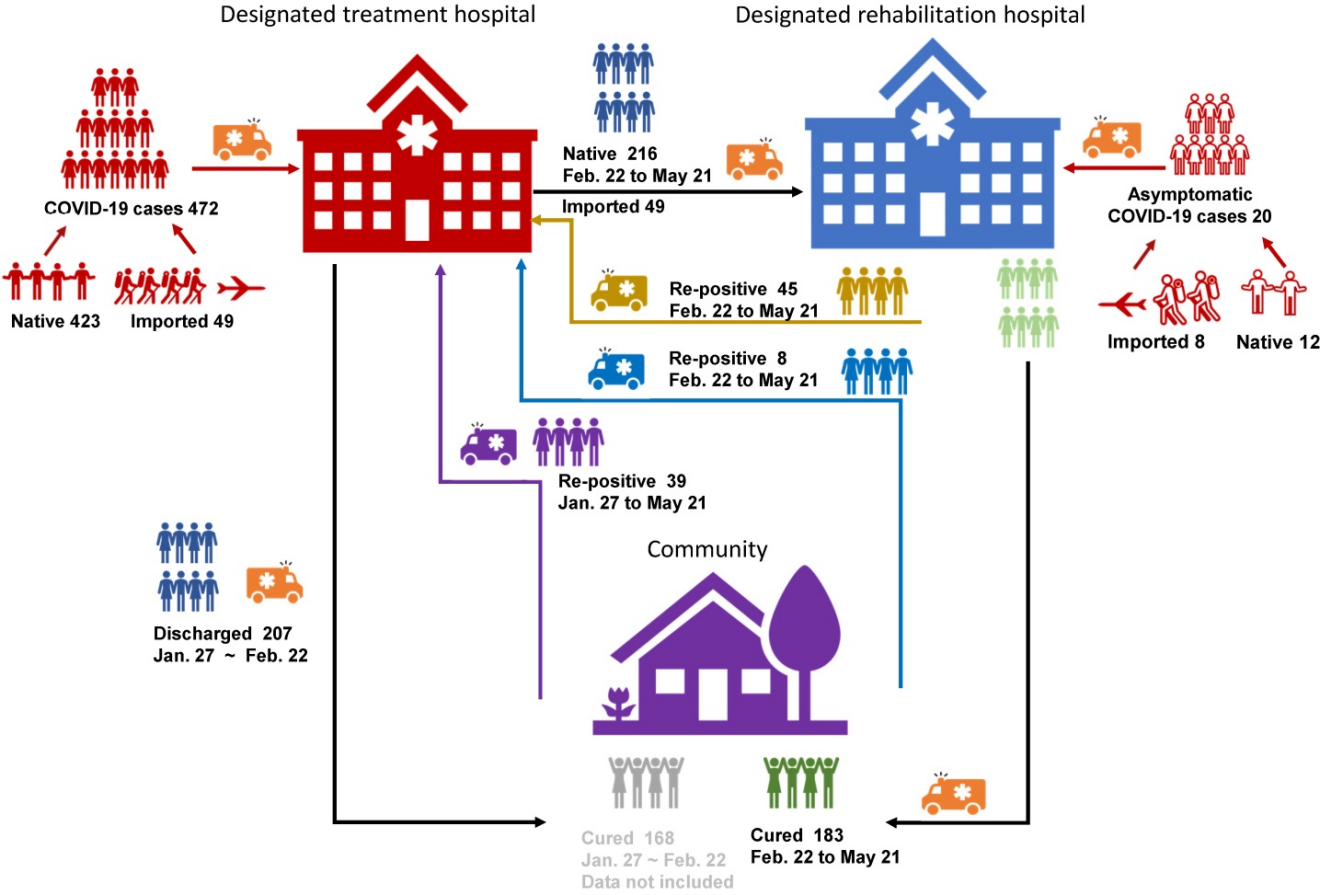
** Cohort information.** There were 472 confirmed CoVID-19 patients (423 native and 49 imported) and 20 asymptomatic carriers (12 native and 8 imported) in total. 207 of the 472 were discharged before February 21^st^ (line black), 168 of the 207 were non-re-positives and were not involved in the study (line Grey).There were 92 re-positives in total, 45 of them turned re-positive during medical observation (line yellow), 8 of them turned re-positive at community test after medical isolation observation (line blue), and 39 cases discharged before February 21^st^ and found re-positive in community test (line violet).

Until 11 May, there were 324 patients under medical isolation, including 304 confirmed patients and 20 asymptomatic ones (Figure 1); average hospital stay of Group A was 19.25 ± 14.79 days, much shorter than that of Group M (24.43 ± 8.62 days) and Group S (33.75 ± 10.28 days). In all, there were 155 men and 169 women. In Group S, there were more men (59.02%) than women (40.98%) (Table 1). The youngest patient was 4 months old, and the eldest one was 86 years old; the average age of Group A (23.44 ± 13.30 years) was lower than that of other groups, and 30% of this group was under 18 years. All members of Group S were above 18 years, mainly elderly, as their average age was 57 ± 11.78 years (Table 1) (Figure 3). Furthermore, 83.61% patients of Group S had some form of comorbidity, and this rate was remarkably higher than that of Group M (48.21%) and Group A (35%); 26.23% of Group S had hypertension, 21.31% had diabetes and 29.51% had cardiovascular diseases, and all rates were much higher than the ones reported in Group A and Group M (Table S1).

During recovery, a few patients developed fever (3 in Group M and 1 in Group S), and the fever subsided after 1 day. In Group S, 20% had chest discomfort, and their CT findings showed a total score of 14 ± 6.49, much higher than that of Group M (5.17 ± 3.51). Group S had higher rates of consolidation and ground glass opacity (GGO) occurrence than those of Group M. This contrast showed that patients with severe and critical symptoms had severe lung damage, even in recovery, and that they needed a longer recovery period and extra care (Table 1, Figure S1).

COVID-19 patients during recovery were likely to have anxiety (11.94% in Group M and 10% in Group S) and headache/insomnia (8.96% in Group M and 22.5% in group S), indicating that they were having mental and psychological problems. Asymptomatic carriers stated having no symptoms during recovery; there was no significant difference among the three groups in terms of abnormalities in blood components related to infection suppression (AST, ALT, PCT, WBC, neutrophil and lymphocyte) (Table 1).

### Ageusia and anosmia during disease and recovery

By random call-back visit to imported cases, we found that some of them had ageusia and anosmia. This was not listed in our earlier random call-back content, and we added this question back to the list and got 38 valid responses from imported patients and 35 from local patients. In all, 10 of the 38 (26.32%) patients claimed to have ageusia/anosmia during the disease course and 5 (13.16%) had ageusia/anosmia during recovery. On the contrary, only 1 (2.86%) local patient had ageusia/anosmia during the disease course, and 7 (20%) patients had ageusia/anosmia during recovery (Figure S2).

### Epidemiological and clinical features of re-positive patients

There were totally 492 patients, with 423 local confirmed cases 10 and 49 imported cases, where 20 were asymptomatic carriers (12 local cases and 8 imported cases). Ninety-two out of 492 patients turned re-positive, showing a re-positive rate of 18.70%. Only 324 patients had been through medical isolation/observation and were involved in this study (Figure 1).

Re-positive (RP) patients had a shorter hospital stay (22.82 ± 9.99 days) than non-re-positive (NRP) patients (26.90 ± 10.12 days), and the time from onset to first negative conversion was shorter for RP patients than for NRP patients (25.78 ± 10.71 days and 30.33 ± 11.11 days, respectively). Time from onset to last negative conversion was longer for RP patients than for NRP patients (59.22 ± 11.07 days and 30.33 ± 11.11 days, respectively); the longest recorded time was 97 days (Table 2). A significantly higher number of women (60.87%) turned re-positive than did men (39.13%, P < 0.05).

**Figure 2:**
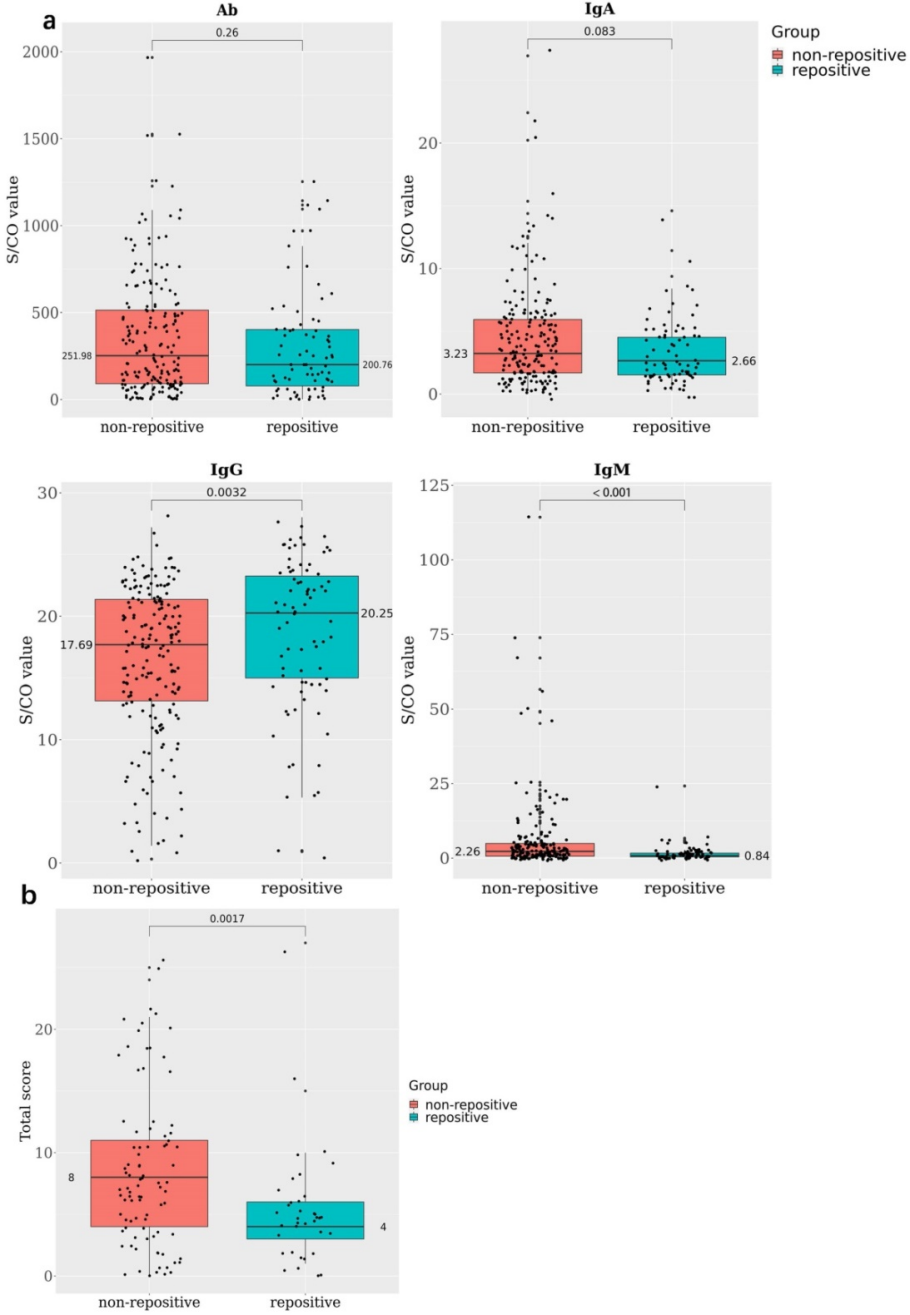

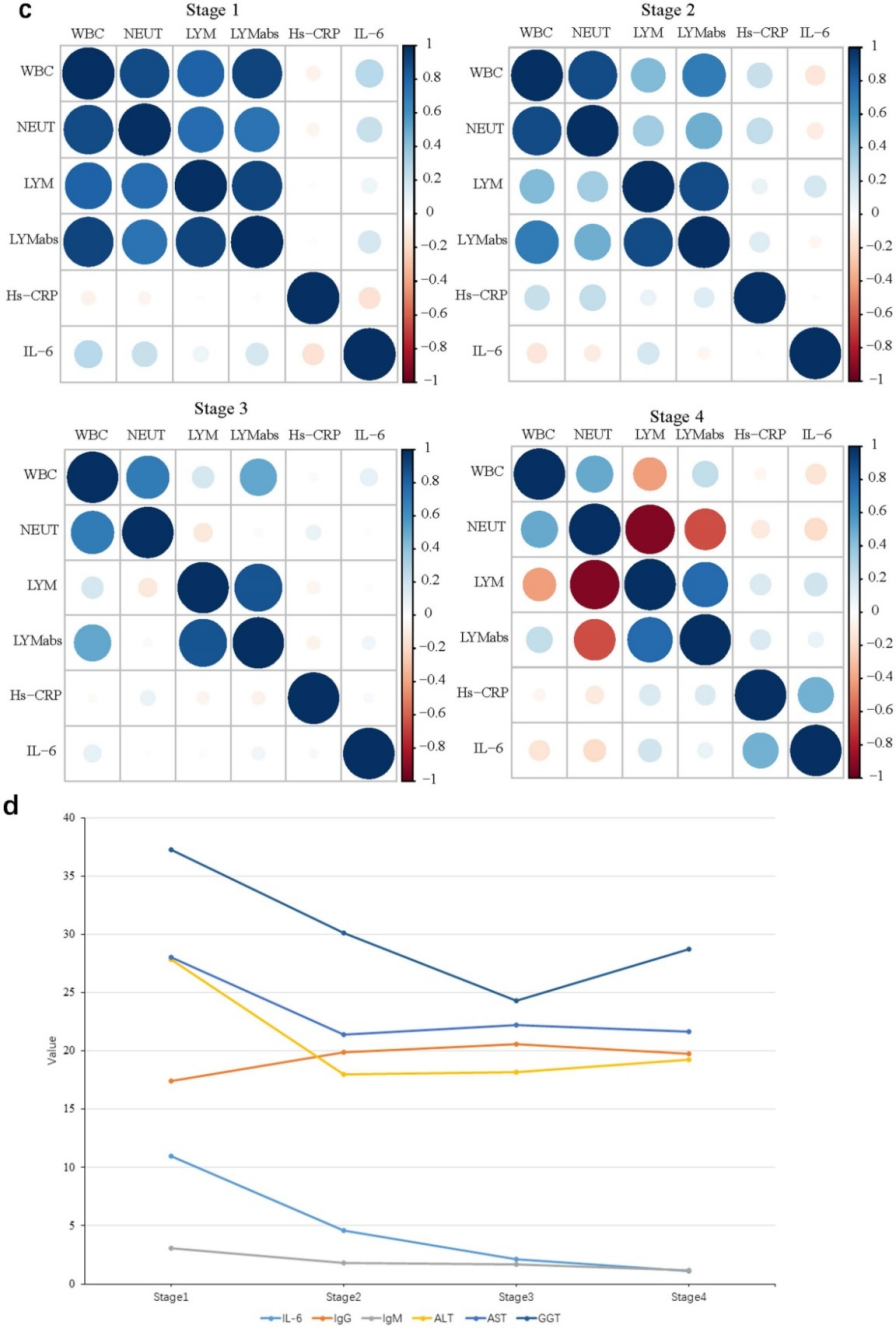
** Antibody and blood biochemical indexes changed in re-positive patients during recovery A.** The comparison of antibody levels against SARS-CoV-2 among 190 none re-positive recovered COVID-19 patients and 78 re-positives, the average S/CO values of total Ab, IgA, IgG, and IgM were compared between re-positives (green) and none re-positives (red). **B.** The comparison of chest CT score among 89 none re-positive recovered COVID-19 patients and 37 re-positives; the average total scores were compared between re-positives (green) and none re-positives (red). **C.** Correlations among WBC, neut, lym, and lym abs during stages 1 to 4. **D.** The trend line of antibody and blood biochemical indexes during the four stages.

**Figure 3:**
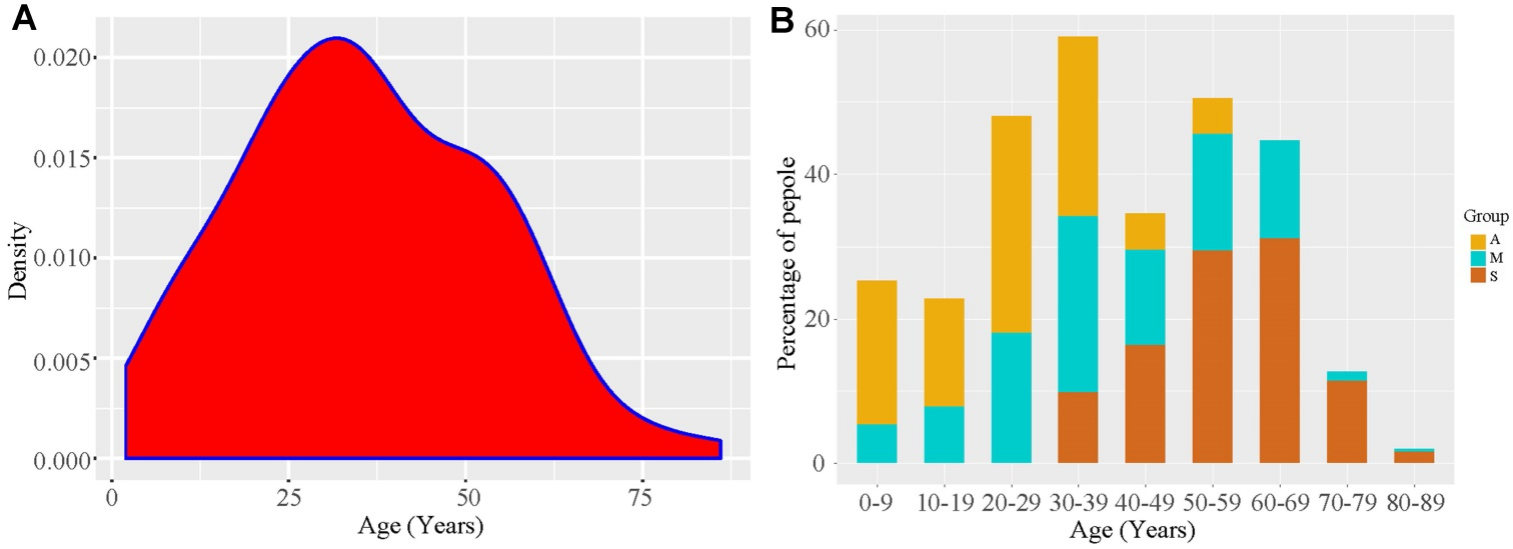
** Patient age distribution. A.** The kernel density plot based on age distribution data. **B.** COVID-19 proportion taken by different types of patients in different age stages. A, asymptomatic carriers; M, mild and moderate cases; S, severe /critically ill cases.

We advised the recovered patients to undergo repeat CT, and 134 patients took the advice; they underwent the test 14+ days after discharge from the hospital. The results showed that the total score of RP patients was 5.22 ± 4.72, much lower than that of NRP patients, i.e., 8.53 ± 6.18; 43; the GGO was 4% for RP patients and 76.40% for NRP patients (Table 2, Figure 2B). This means that RP patients had less severe lung disorder than NRP patients. At the same time, we found the average age of RP patients was much lower than that of NRP patients (34.5 ± 17.45 vs 43.5 ± 18.49), and RP patients had fewer comorbidities than did NRP patients (30.43% vs 53.44%) (Table S2). The symptoms during recovery showed no obvious difference between RP and NRP patients (Table 2). Furthermore, patients who had taken antibacterial agents, immunoregulatory drugs, and acetylcysteine during the disease course had low re-positive rates (Table S2); there was no significant difference between RP and NRP patients in inflammation-related indicators or antibodies.

### Antibody and blood biochemical index changes in RP patients during recovery

In all, 78 RP and 190 NRP patients conducted antibody detection tests; 46.15% of the RP and 66.32% of the NRP patients were positive for IgM, 98.72% of the RP and 99.47% of the NRP patients were positive for IgG, and all patients in both RP and NRP groups were positive for total antibody (Ab). The S/COI of IgG was remarkably higher in the RP group than in the NRP group (20.25 and 17.69, respectively, P < 0.005) (Figure 2A).

Forty-one patients stayed in medical isolation observation for more than 2 months owing to repeated positive nucleic acid findings; they went through four stages of antibody and blood biochemistry tests (Figure S3). All IgG tests showed positive results, and IgM-positive rates decreased from 57.9% in the first stage to 23.7% in the fourth stage (Table S3). Meanwhile, the WBC, neut, lym%, and lym abs were positively correlated at stage 1, and the positive correlations among them decreased in stages 2 and 3 and turned to negative in stage 4 (Figure 2C). At the same time, the IL-6 value decreased gradually at each stage; this means the inflammatory response of RP patients were falling to normal levels. Indicators related to liver functions such as ALT, AST, and γ-glutamyl transpeptidase (GGT) rapidly levelled out (stage 1 and 2) and stabilized (stage 3 and 4); this showed that the damaged liver functions were recovering (Figure 2D).

## Discussion

Asymptomatic carriers were mainly young patients aged under 35 years, where 30% of them were children under 18 years old, while patients with severe/critical symptoms were mainly older; 59.2% of the severely ill were men, which is quite different from that in a previous study 11, showing no significant differences between the sexes. Older patients with comorbidities had worse symptoms than young patients, especially on hypertension, diabetes and cardiovascular diseases; the incidence rate of group S was significantly higher than that of group A and M, which is similar with other studies 12,13. Meanwhile, we found that older patients had a lower RP rate, and this may be due to the increased number of antiviral and immunoregulatory drugs used and long hospital stay (Table 1). During the recovery period, most patients showed chest discomfort and headache/insomnia, especially those with severe symptoms; chest CT images also showed that severely ill patients had worse chest symptoms and higher scores (Figure S1) than moderately ill patients. Thus, even in recovery, infection in lungs and mental health problems should be given attention.

In our previous study, 42 of 96 had stated on their own account that they had mild depression 14; Ageusia and anosmia during COVID-19 disease period were believed to be related to ACE2 and TMPRSS2 expression on epithelial cells 15, but in this study, we found those symptoms occurred during the recovery period, probably related to mental conditions 16. These points indicate that the mental and psychological health of these patients must be tracked.

Asymptomatic carriers did not show any symptoms during recovery nor did their antibody or inflammatory indicator levels show any abnormality. Laboratories in Wuhan tried to culture the cells in the sputum of 300+ asymptomatic carriers, but they failed to culture any live virus 17, indicating that asymptomatic carriers had very low infectiousness if they were infectious at all.

Among all the patients, 18.7% showed positive nucleic acid results, slightly higher than that in other studies 18,19. There were 92 RP patients in total including 4 asymptomatic carriers (20% of Group A), 11 patients with severe/critical symptoms (18.03% of Group S), and 77 patients with mild symptoms (31.69% of Group M). The average hospital stay of RP patients was 3.64 days shorter than that of NRP patients; the time from onset to discharge for RP patients was 5.21 days shorter than that for NRP patients. On average, RP patients were 9 years younger than NRP patients. Women had higher RP rates than men.

The CT images of recovering patients showed that RP patients had fewer lung lesions and better prognosis than NRP patients, while symptoms and laboratory testing results during disease and recovery periods showed no major difference between RP and NRP patients. It was reported that 40% of asymptomatic carriers and 12.9% of patients with different symptoms showed negative results on antibody detection 8 weeks after discharge 20. While in this study, we found no negative IgG result for two months in the antibody detections for the 41 repeat nucleic acid-positive patients, this might due to the high IgG expression triggered by the remaining viral RNA. Viral load was believed to be connected with disease progression, but in these cases, their inflammatory indicators were at the normal levels (Table 2, Table S3) and had no remarkable difference from those in the NRP group. Thus, we believe that the positive RT-PCR result of the RP group was evidence of viral shedding and virus not reproducing. Cultured cells of RP patients failed to yield any live SARS-CoV-2 virus 21, indicating that RP patients were not progressing in the disease course.

Furthermore, antibacterial agents, immunoregulatory drugs, and acetylcysteine taken during the disease period had affected the RP rates because RP samples were mainly taken from the nasopharyngeal swab 8, and the stated drugs are helpful in enhancing immunity and eliminating phlegm; this further helped eliminate the virus, especially those in the upper respiratory tract.

Regarding the reasons for RP results, some reports stated there were false-negative RT-PCR results on the discharge test 22. In our current study, some patients showed a positive result during the 40-49 days after discharge, and they had gone through 5-7 rounds of tests. Considering the rate of false-negative results, we believe it is very unlikely to have false-negative results so many times consecutively. According to Young et al., RT-PCR tests for SARS-COV-2 patients randomly show negative results 23, possibly because COVID-19 patients have long-term intermittent viral shedding during recovery.

The longest viral shedding period we have observed was 97 days, and the longest interval between two positive results were 48 days (Table 2); this record can be broken as we extend the follow-up period. Based on this assumption, quarantining RP patients could be unpractical, at designated medical centres or at home. Fortunately, the RP patients showed no evidence of disease progression or infectivity, so we suggest that CT imaging results and clinical manifestations should be considered and referred during the follow-up of RP.

This study has several limitations. Firstly, it was a single-center retrospective study, and multiple-centers clinical observations would be more scientific and reliable to evaluate the physiological, mental, and social health of COVID-19 recovered patients. Secondly, the conclusion could be biased by the small size of samples. A larger scale of research is necessary for evaluating the clinical, viral, and immunological characteristics of asymptomatic carriers in the future. Further, the follow-up time span was too short and a longer period of follow-up study should be performed.

## Conclusion

During recovery, COVID-19 patients need care and proper therapy for both mental and psychological health, and severely ill patients need an extended period of time for recovery. Re-positivity can occur in any person, regardless of age, sex, or clinical symptoms, but juveniles, females, and patients with mild/moderate existing symptoms have higher rates of re-positivity than their adult, male, and severe/critical counterparts, respectively. Although there is no evidence that turning re-positive has an impact on their infectivity, CT imaging results and clinical manifestations have alerted us that we need differentiate them in the following managements.

## Supplementary Material

Supplementary figures and tables.

## Figures and Tables

**Table 1:** Clinical characteristics of recovered COVID-19 patients

Total (n=324)	Asymptomatic cases (n=20)	Mild/Moderate cases (n=243)	Severe/Critical cases (n=61)	P1	P2	P3
Local cases (n=267)	12	195	60			
Imported cases (n=57)	8	48	1			
Average hospital stay (days)	19.25±14.79	24.43±8.62	33.75±10.28	<0.001	<0.001	<0.001
Number of re-positive cases	4 (20)	77 (31.69)	11 (18.03)	0.278	0.852	<0.05
**Gender, n**	20	243	61	0.983	0.279	0.055
Male, n (%)	9 (45)	110 (45.27)	36 (59.02)			
Female, n (%)	11 (55)	133 (54.73)	25 (40.98)			
**Age, n**	20	243	61			
Median age (years)	24 (IQR 11 months-38 years)	37 (IQR 4 months-81 years)	58 (IQR 31-86)			
Average age (years)	23.44±13.30	38.61±17.71	57±11.78	<0.001	<0.001	<0.001
≤18 years, n (%)	6 (30)	26 (10.7)	0	0.411	-	-
>18 years, n (%)	14 (70)	217 (89.3)	61 (100)	<0.01	<0.001	<0.001
**Convalescent symptoms, n**	10 (%)	134 (%)	40 (%)			
Fever, n=4	0	3 (2.24)	1 (2.5)	0.645	0.653	0.929
Cough/stuffy nose, n=9	0	7 (5.22)	2 (5)	0.467	0.497	0.959
Chest distress/shortness of breath, n=15	0	7 (5.22)	8 (20)	0.467	0.131	<0.01
Anxious/depression, n=20	0	16 (11.94)	4 (10)	0.251	0.314	0.739
Skin pruritus, n=5	0	4 (2.99)	1 (2.5)	0.59	0.653	0.877
Headache/insomnia, n=21	0	12 (8.96)	9 (22.5)	0.329	0.105	<0.05
**Laboratory test, n**	15 (14)	196 (173)	52 (52)			
Elevated AST, n (%)	0	20/196 (10.02)	6/52 (11.54)	0.196	0.176	0.782
Elevated ALT, n (%)	1/15 (6.67)	17/196 (8.67)	6/52 (11.54)	0.793	0.599	0.529
Elevated PCT, n (%)	2/14 (14.29)	14/173 (8.09)	3/52 (7.32)	0.43	0.449	0.872
Elevated WBC, n (%)	1/14 (7.14)	1/173 (0.58)	0	<0.05	0.095	0.635
Elevated LYM%, n (%)	1/14 (7.14)	5/173 (2.89)	2/52 (4.88)	0.391	0.768	0.524
Elevated NEUT%, n (%)	0	1/173 (0.58)	0	0.792	/	0.635
**Chest CT, n (%)**	0	92	34	/	/	
Total scores	/	5.17±3.51	14±6.49	/	/	<0.001
GGO, n (%)	/	51 (55.43)	33 (97.06)	/	/	<0.001
Consolidation, n (%)	/	11/92 (11.96)	13/34 (38.24)	/	/	<0.001
Crazy-paving sign, n (%)	/	0/92 (0)	3/34 (8.24)	/	/	<0.01

p1, p2, and p3 were comparisons between asymptomatic patients and those with mild/moderate disease, asymptomatic and severely/critically ill patients, and those with mild/moderate disease and severely/critically ill patients, respectively. All data were analysed using the Mann-Whitney U test. P values <0.05 indicate significant differences.

**Table 2:** Clinical characteristics of re-positive COVID-19 patients

	Re-positives (n=92)	Non-re-positives (n=232)	P-Value
Total (n=324)	92	232	
Symptomatic cases (n=20)	4	16	0.198
Mild/moderate cases (n=243)	77	166	
Severe/critically cases (n=61)	11	50	
Average hospital stays (days)	22.82±9.99	26.90±10.12	<0.01
Days from onset to discharge	25.78±10.71	30.33±11.11	<0.001
Days from discharge to last RNA-negative conversion	59.22±11.07 (IQR 17-97)	30.33±11.11	<0.001
Interval between two positive results (days)	13.54±10.47 (IQR 2-48)	/	
**Gender, n**			<0.05
Male	36 (39.13)	119 (51.29)	
Female	56 (60.87)	113 (48.71)	
**Age**			
Median age (years)	35 (IQR 0.92-86)	43 (IQR 0.33 -81)	
Average age (years)	35.95±17.45	47.12±16.47	<0.001
≤18 years, n (%)	14 (15.22)	18 (7.76)	0.203
>18 years, n (%)	78 (84.78)	214 (92.24)	<0.01
Chest CT imaging, n	37	89	
Total scores	5.22±4.72	8.53±6.18	<0.01
GGO, n (%)	16 (43.24)	68 (76.40)	<0.01
Consolidation, n (%)	4 (10.81)	20 (22.47)	0.132
Crazy-paving sign, n (%)	1 (2.70)	2 (2.25)	0.887
**Convalescent symptoms, n**	62	122	
Fever, n (%)	1 (1.61)	3 (2.46)	0.715
Cough/stuffy nose, n (%)	3 (4.84)	6 (4.92)	0.984
Chest distress/shortness of breath, n (%)	3 (4.84)	12 (9.84)	0.244
Anxious/depression, n (%)	8 (12.90)	12 (9.84)	0.53
Skin pruritus, n (%)	1 (1.61)	4 (3.28)	0.516
Headache/insomnia, n (%)	8 (12.90)	13 (10.66)	0.653
**Laboratory test, n**	68 (57)	194 (170)	
Elevated AST, n (%)	8/68 (11.76)	18/194 (9.28)	0.557
Elevated ALT, n (%)	9/68 (13.24)	15/194 (7.73%)	0.177
Elevated PCT, n (%)	6/57 (10.53)	13/170 (7.65%)	0.499
Elevated WBC, n (%)	0	2/170 (1.18%)	0.416
Elevated LYM%, n (%)	2/57 (3.51%)	6/170 (3.53%)	0.997
Elevated NEUT%, n (%)	1/57 (1.75%)	0	0.086
**Antibody/inflammatory cytokines, n**	78	190	
Positive IgM, n (%)	36 (46.15)	126 (66.32)	<0.01
Negative IgG, n (%)	1 (1.28)	1 (0.53)	<0.05
Elevated Hs-CRP, n (%)	0	0	<0.01
Elevated SAA, n (%)	4 (5.13)	12 (6.32)	0.771

All data were analysed using the Mann-Whitney U test. P values <0.05 indicate significant differences.
