# Supplementary material for: Clinical characteristics and follow-up analysis of 324 discharged COVID-19 patients in Shenzhen during the recovery period
Source: Int J Med Sci. 2021 Jan 1;18(2):347–55. doi: 10.7150/ijms.50873 (PMC7757147; doi:10.7150/ijms.50873)

Supplementary information

Supplementary methods

Cohort Information

Discharge criteria according to “Diagnosis and Treatment of Pneumonia Caused by Novel Coronavirus (Trial Version 7): (1) Temperature below 37 degrees Celsius lasting at least 3 consecutive days, (2) resolved respiratory symptoms, (3) substantial improvement in chest lesions in computed tomography (CT) images, and (4) two consecutively negative RT-PCR test results with at least 1-day interval.

This study included patients who satisfied the following criteria:

(1) Patients who reached the above discharge criteria, (2) asymptomatic carriers who showed negative results after treatment, (3) patients discharged before February 21st and showing positive results on re-test in the 2-week routine tests, (4) imported cases with positive results on nucleic acid tests or those who claimed to have confirmed COVID-19. We categorized the patients into three categories: Asymptomatic carriers, mildly/moderately ill patients, and severely/critically ill patients. The classification is in Diagnosis and Treatment of Pneumonia Caused by Novel Coronavirus (Trial Version 7). In brief, the mild type has no signs of pneumonia on chest imaging; the moderate type includes fever and respiratory symptoms, and signs of pneumonia on radiologic assessment; the severe type meets any of the following criteria: 1) shortness of breath, RR ⩾ 30 times/min; 2) oxygen saturation ⩽ 93% at rest; 3) arterial oxygen partial pressure/fraction of inspiration O2 (PaO2/FiO2 ⩽ 300 mmHg); and 4) pulmonary imaging showing significant progression of lesion >50 % within 24 - 48 hours. The severe type includes 1) respiratory failure and requiring mechanical ventilation, 2) shock or 3) with other organ failure that requires ICU care. “Nucleic acid re-positive” means the patients’ nasopharyngeal swabs/anal swabs were found to be positive again after reaching the discharge criteria and being discharged from the hospital.

Glossary

Imported cases: Those who were tested at customs entry points and found to be nucleic acid positive or confirmed overseas and discharged after treatment, or confirmed, treated, and discharged in other cities in China. The number of confirmed imported cases is different from the official reported number, as only patients confirmed for the first time in Shenzhen were reported.

Average hospital stay: Days between first admission to hospital and discharge. Repeated hospitalization periods due to re-positive testing results are not counted.

Days from onset to negative conversion: Days between the onset as per the patient's own account and the day of discharge from the hospital.

Days from onset to last negative conversion: Days between onset as per patient's own account and last date when the patient showed a positive result. Concerning asymptomatic carriers, we recorded the first day when the patient showed a positive result on testing as the onset day.

Days from discharge to last RNA negative conversion: Days between the discharge day and the last date when the patient showed a positive result.

Interval between two positive results: Days between the discharge day and first day when a positive result was found.

Convalescent symptoms: Observed during medical isolation and after discharge.

RT-PCR Analysis

The real time reverse transcription-polymerase chain reaction (RT-PCR) tests were performed with nasopharyngeal and anal swabs by Shenzhen Center for Disease Control and Prevention (CDC) using the High Pure Viral RNA Kit (Roche, Mannheim, Germany) and 2019n-CoV Viral RNA detection kit (Bio-Germ, Shanghai, China). RNA was extracted from a collection tube of nasopharyngeal swabs/anal swabs with 1.5 mL of virus preservation solution. Then, 200 μL of cell lysate was taken for 10 s vortex followed by 10 min at room temperature. Then, the suspension was collected after 10 min of centrifugation at 1, 000 rpm/min. Two target genes of SARS-COV-2, including open reading frame 1ab (ORF1ab) and nucleocapsid protein (N), were simultaneously

amplified and tested during RT-PCR assay. Target 1 (ORF1ab): forward primer CCCTGTGGGTTTTACACTTAA; reverse primer ACGATTGTGCATCAGCTGA; and the probe 5′-VIC-CCGTCTGCGGTATGTGGAAAGGTTATGG-BHQ1-3′. Target 2 (N): forward primer GGGGAACTTCTCCTGCTAGAAT; reverse primer CAGACATTTTGCTCTCAAGCTG; and the probe 5′-FAM-TTGCTGCTGCTTGACAGATT-TAMRA-3′. A cycle threshold value (Ct value) less than 37 was defined as positive, and Ct value no less than 40 was defined as negative. A medium load, more than 37 and less than 40, will be defined as weak positive, which would require further confirmation by retesting. If Ct value was ≤40 in the re-test next day, a positive result would be reported.

Antibody Detection and Laboratory Testing

The main results and indicators of epidemiology, demography, clinical manifestation, and laboratory examination of 182 recovered patients with COVID-19 were collected and analysed. The inflammation markers and liver function-related factors were tested, including interleukin-6 (IL-6), procalcitonin (PCT), white blood cell (WBC) count, absolute value of lymphocyte (lym abs), percentage of lymphocytes (lym%), percentage of neutrophils (neut%), hypersensitive-c-reactive-protein (Hs-CRP), alanine aminotransferase (ALT), aspartate aminotransferase (AST), and gamma glutamine transferase (GGT). The total antibody (Ab), IgA, IgG, and IgM levels were tested on the seventh day with SARS-CoV-2 testing kit (WANTAI BioPharm, Beijing, China) using the chemiluminescence method. All the tests were performed according to the manufacturer’s instructions. An S/CO <1 indicated a negative result, and S/CO ≥1 indicated a positive result.

Supplementary tables

Table S1 Clinical characteristics of recovered COVID-19 patients during disease period

Total (n=324)	Asymptomatic cases (n=20)	Mild/Moderate cases (n=243)	Severe/Critical cases (n=61)	P1	P2	P3
Local cases (n=267）	12	195	60			
Imported cases (n=57)	8	48	1			
**Comorbidity, n (%)**	7 (35)	94 (48.21)	51 (83.61)	0.262	<0.001	<0.001
Hypertension, n (%)	1 (5)	25 (10.29)	16 (26.23)	0.449	<0.05	0.001
Diabetes, n (%)	0	12 (4.94）	13 (21.31)	0.312	0.027	<0.001
Hyperlipemia, n (%)	1 (5)	4 (1.65）	0	0.295	0.086	0.316
Cardiovascular disease, n (%)	0	19 (7.82)	18 (29.51)	0.196	<0.05	<0.001
Malignant tumor, n (%)	0	3 (1.23)	2 (3.28)	0.624	0.427	0.264
**Treatment, n**	15	227	61			
Antiviral drugs, n (%)	13 (87)	220 (96.92)	60 (98.36)	<0.05	<0.05	0.545
Antimicrobial drugs, n (%)	1 (7)	35 (15.42)	36 (59.02)	0.359	<0.001	<0.001
Immunomodulatory drugs, n (%)	0	63 (27.75)	50 (81.97)	<0.05	<0.001	<0.001
Acetylcysteine, n (%)	0	58 (25.55)	32 (52.46)	<0.05	<0.001	<0.001
Traditional Chinese medicine, n (%)	4 (27)	84 (37)	21 (34.43)	0.422	0.575	0.712
Live bacterium tablet, n (%)	3 (20)	78 (34.36)	30 (49.18)	0.256	<0.05	<0.05

p1, p2, and p3 were comparisons between asymptomatic patients and patients with mild/moderate disease, asymptomatic and severely/critically ill patients, and patients with mild/moderate cases disease and severely/critically ill patients, respectively. All data were analysed using the Mann–Whitney *U* test. P values <0.05 indicate significant differences.

Table S2 Clinical characteristics of re-positive COVID-19 patients during disease period

	Re-positives (n=92)	Non-re-positives (n=232)	P-Value
Total (n=324)	92	232	
Symptomatic cases (n=20)	4	16	
Mild/moderate cases (n=243)	77	166	0.198
Severe/critically cases (n=61)	11	50	
**Comorbidity, n (%)**	28/92 (30.43)	124/232 (53.44)	<0.001
Hypertension, n (%)	8 (8.70)	34 (14.66)	0.151
Diabetes, n (%)	4 (4.35)	21 (9.05)	0.153
Hyperlipemia, n (%)	3 (3.26)	2 (0.86)	0.115
Cardiovascular disease, n (%)	7 (7.61)	30 (12.93)	0.175
Hepatopathy, n (%)	5 (5.43)	31 (13.36)	<0.05
**Treatment, n**	90	212	
Antiviral drugs, n (%)	88 (97.78)	205 (96.70)	0.616
Antimicrobial drugs, n (%)	20 (22.22)	72 (33.96)	<0.05
Immunomodulatory drugs, n (%)	25 (27.78)	88 (41.51)	<0.05
Acetylcysteine, n (%)	17 (18.89)	73 (34.43)	<0.01
Traditional Chinese medicine, n (%)	28 (31.11)	81 (38.21)	0.241
Live bacterium tablet, n (%)	29 (32.22)	82 (38.68)	0.288

All data were analysed using the Mann–Whitney *U* test. P values <0.05 indicate significant differences.

Table S3 Laboratory test of 41 re-positive patients

/	Stage	N	average value	SD	P1	P2	number of outliers	number of normal values	Ratio of outliers（%）
interval between the current test to the discharge day	1	41	20.5	15.7	/	/	/	/	/
	2	41	30.4	12.0	0.001	/	/	/	/
	3	41	36.4	12.4	0.000	0.048	/	/	/
	4	41	42.6	13.5	0.000	0.039	/	/	/
TP (g/L) reference: 65-85	1	41	73.0	5.2	/	/	1	40	2.4
	2	41	73.0	3.5	0.994	/	0	41	0.0
	3	41	72.8	4.4	0.848	0.853	3	38	7.3
	4	41	73.8	5.1	0.416	0.315	1	40	2.4
ALB(g/L) reference: 35-52	1	41	45.6	3.2	/	/	1	40	2.4
	2	41	46.1	3.0	0.430	/	1	40	2.4
	3	41	46.4	2.9	0.199	0.619	1	40	2.4
	4	41	46.9	3.1	0.048	0.481	2	39	4.9
GLB (g/L) reference: 20-40	1	41	27.4	4.7	/	/	1	40	2.4
	2	41	26.9	3.9	0.585	/	1	40	2.4
	3	41	26.4	4.6	0.281	0.595	3	38	7.3
	4	41	26.9	4.4	0.607	0.573	2	39	4.9
ALB/GLB reference: 1.5-2.5	1	41	1.7	0.3	/	/	7	34	17.1
	2	41	1.7	0.3	0.440	/	6	35	14.6
	3	41	1.8	0.4	0.070	0.295	9	32	22.0
	4	41	1.8	0.3	0.218	0.557	7	34	17.1
PA (g/L) reference: 0.2-0.4	1	41	0.3	0.1	/	/	2	39	4.9
	2	41	0.3	0.0	0.476	/	0	41	0.0
	3	40	0.3	0.0	0.493	0.982	0	40	0.0
	4	41	0.3	0.0	0.740	0.311	0	41	0.0
TBA (μmol/L) reference: 0-10	1	41	8.0	6.9	/	/	10	31	24.4
	2	41	8.5	5.6	0.655	/	13	28	31.7
	3	41	7.9	4.4	0.983	0.640	12	29	29.3
	4	41	7.6	3.6	0.766	0.782	11	30	26.8
ALT (U/L) reference: Female 7-40; male 9-50	1	41	27.9	37.5	/	/	8	33	19.5
	2	41	18.0	12.8	0.046	/	10	31	24.4
	3	41	18.1	13.5	0.050	0.969	11	30	26.8
	4	41	19.2	15.1	0.081	0.826	4	37	9.8
AST (U/L) reference: 15-40	1	41	28.0	26.7	/	/	10	31	24.4
	2	41	21.4	9.1	0.054	/	8	33	19.5
	3	41	22.2	9.5	0.092	0.806	7	34	17.1
	4	41	21.7	8.6	0.065	0.873	3	38	7.3
	1	41	1.3	0.7	/	/	11	30	26.8
	2	41	1.5	0.7	0.172	/	15	26	36.6

AST/ALT reference: 0.5-1.5	3	41	1.6	0.8	0.062	0.614	18	23	43.9
	4	41	1.4	0.7	0.606	0.175	11	30	26.8
GGT (U/L) reference: female 7-45; male 10-60	1	41	37.2	33.7	/	/	8	33	19.5
	2	41	30.1	28.3	0.209	/	3	38	7.3
	3	41	24.3	16.4	0.024	0.308	4	37	9.8
	4	41	28.7	20.8	0.135	0.435	3	38	7.3
ALP (U/L) reference: 40-130	1	41	32.0	20.5	/	/	30	11	73.2
	2	41	21.1	14.7	0.003	/	36	5	87.8
	3	41	23.6	18.4	0.022	0.502	32	9	78.0
	4	41	19.9	10.1	0.001	0.318	39	2	95.1
TC (mmol/L) reference: 0-5.2	1	41	5.0	1.1	/	/	16	25	39.0
	2	41	4.9	1.2	0.749	/	13	28	31.7
	3	41	5.1	1.1	0.702	0.482	18	23	43.9
	4	40	5.3	1.1	0.215	0.389	22	18	55.0
TG (mmol/L) reference: 0-2.6	1	41	2.3	1.1	/	/	13	28	31.7
	2	41	2.1	0.9	0.459	/	9	32	22.0
	3	41	2.4	1.3	0.646	0.231	13	28	31.7
	4	40	2.3	1.2	0.948	0.696	11	29	27.5
ApoA1 (g/L) reference: female 1.08-2.25; male 1.04-2.02	1	41	1.4	0.2	/	/	1	40	2.4
	2	41	1.4	0.2	0.308	/	1	40	2.4
	3	41	1.5	0.2	0.246	0.886	1	40	2.4
	4	40	1.5	0.2	0.106	0.642	1	39	2.5
ApoB (g/L) reference: female 0.60-1.17; male 0.66-1.33	1	41	0.9	0.2	/	/	6	35	14.6
	2	41	0.9	0.3	0.825	/	10	31	24.4
	3	41	0.9	0.2	0.339	0.462	8	33	19.5
	4	40	1.0	0.2	0.038	0.257	8	32	20.0
ApoA1 /ApoB reference: 0.8-2.2	1	41	1.7	0.5	/	/	5	36	12.2
	2	41	1.8	0.6	0.442	/	8	33	19.5
	3	41	1.7	0.5	0.889	0.529	7	34	17.1
	4	40	1.6	0.4	0.409	0.335	5	35	12.5
HDL-C (mmol/L) reference: 1-1.55	1	41	1.2	0.3	/	/	14	27	34.1
	2	41	1.2	0.3	0.875	/	14	27	34.1
	3	41	1.2	0.3	0.812	0.936	15	26	36.6
	4	40	1.2	0.3	0.521	0.685	13	27	32.5
LDL-C (mmol/L) reference: 1.9-3.1	1	41	3.0	0.9	/	/	17	24	41.5
	2	41	3.0	1.1	0.956	/	20	21	48.8
	3	41	3.1	1.0	0.826	0.783	21	20	51.2
	4	40	3.3	1.1	0.192	0.276	23	17	57.5
LP(a) (mg/dL) reference: 0-30	1	41	25.8	27.3	/	/	12	29	29.3
	2	41	22.4	24.4	0.564	/	11	30	26.8
	3	41	22.7	26.8	0.594	0.965	11	30	26.8

	4	40	26.2	28.5	0.955	0.558	11	29	27.5
Glu (mmol/L) reference: 3.9-6.1	1	41	5.6	1.3	/	/	15	26	36.6
	2	41	6.0	1.8	0.327	/	19	22	46.3
	3	41	6.2	2.4	0.160	0.669	11	30	26.8
	4	41	6.5	2.4	0.053	0.593	17	24	41.5
LDH (U/L) reference: 135-214	1	41	190.4	37.7	/	/	12	29	29.3
	2	41	166.3	25.1	0.001	/	5	36	12.2
	3	41	163.1	32.6	0.000	0.644	9	32	22.0
	4	41	164.1	27.0	0.000	0.879	10	31	24.4
α-HBDH (U/L) reference: 78-182	1	41	131.6	24.7	/	/	0	41	0.0
	2	41	118.8	20.7	0.017	/	0	41	0.0
	3	41	120.8	27.5	0.043	0.714	3	38	7.3
	4	41	121.6	22.7	0.062	0.873	0	41	0.0
CysC (mg/L) reference: 0-60 years 0.59-1.03; > 60 years 0-1.5	1	41	1.0	0.2	/	/	11	30	26.8
	2	41	1.0	0.3	0.929	/	10	31	24.4
	3	41	1.0	0.2	0.521	0.580	7	34	17.1
	4	41	1.0	0.3	0.989	0.529	9	32	22.0
Urea (mmol/L) reference: 2.78-8.07	1	41	4.4	1.7	/	/	5	36	12.2
	2	41	4.3	1.4	0.714	/	4	37	9.8
	3	41	4.4	1.5	1.000	0.714	5	36	12.2
	4	41	4.6	1.5	0.714	0.714	1	40	2.4
Cr (μmol/L) reference: female 45-84; male 59-104	1	41	60.9	17.8	/	/	7	34	17.1
	2	41	60.4	17.2	0.886	/	11	30	26.8
	3	41	59.5	17.7	0.717	0.827	11	30	26.8
	4	41	58.2	17.8	0.492	0.745	14	27	34.1
UA (μmol/L) reference: female 119-416; male 202.3-416.5	1	41	319.8	88.0	/	/	6	35	14.6
	2	41	317.5	77.3	0.898	/	5	36	12.2
	3	41	318.5	80.3	0.941	0.956	5	36	12.2
	4	41	318.1	83.6	0.923	0.982	5	36	12.2
CO2CP (mmol/L) reference: 21-31	1	41	24.9	2.2	/	/	3	38	7.3
	2	41	24.3	2.6	0.319	/	3	38	7.3
	3	41	24.0	2.3	0.123	0.583	3	38	7.3
	4	41	24.5	2.4	0.452	0.428	5	36	12.2
β2-MG (mg/L) reference: 0.8-2.2	1	41	1.9	0.8	/	/	5	36	12.2
	2	41	1.8	0.8	0.510	/	4	37	9.8
	3	41	1.6	0.9	0.147	0.426	8	33	19.5
	4	41	1.8	0.9	0.540	0.400	5	36	12.2
HCY (μmol/L) reference: 0-60years 0-	1	41	10.7	5.0	/	/	10	31	24.4
	2	41	12.7	4.0	0.030	/	16	25	39.0
	3	41	13.0	3.9	0.013	0.760	13	28	31.7
	4	40	12.8	3.6	0.025	0.827	10	30	25.0

15; >60years 15-20									
WBC (109/L) reference: 3.5-9.5	1	33	5.8	1.3	/	/	0	33	0.0
	2	37	5.7	1.2	0.672	/	1	36	2.7
	3	40	5.7	1.0	0.681	0.983	0	40	0.0
	4	41	5.7	0.9	0.649	0.964	0	41	0.0
NEUT (%) reference: 40-75	1	33	57.6	7.1	/	/	2	31	6.1
	2	37	56.8	10.1	0.706	/	3	34	8.1
	3	40	58.8	8.4	0.560	0.319	2	38	5.0
	4	41	60.0	8.4	0.239	0.533	1	40	2.4
NEUTabs (109/L) reference: 1.8-6.3	1	33	3.4	1.0	/	/	1	32	3.0
	2	37	3.3	1.1	0.799	/	4	33	10.8
	3	40	3.4	0.9	0.934	0.723	2	38	5.0
	4	41	3.5	0.9	0.704	0.755	0	41	0.0
LYM (%) reference: 20-50	1	33	30.0	6.6	/	/	1	32	3.0
	2	37	31.4	8.9	0.448	/	4	33	10.8
	3	40	29.8	7.7	0.922	0.370	4	36	10.0
	4	41	29.2	7.1	0.668	0.728	2	39	4.9
LYMabs (109/L) reference: 1.1-3.2	1	33	1.7	0.5	/	/	5	28	15.2
	2	37	1.7	0.4	0.956	/	1	36	2.7
	3	40	1.7	0.5	0.567	0.595	4	36	10.0
	4	41	1.6	0.4	0.324	0.664	2	39	4.9
MONO (%) reference: 3-10	1	33	9.0	2.3	/	/	11	22	33.3
	2	37	8.5	2.7	0.376	/	9	28	24.3
	3	40	8.2	2.1	0.159	0.599	9	31	22.5
	4	41	8.0	2.1	0.057	0.605	6	35	14.6
MONabs (109/L) reference: 0.1-0.6	1	33	0.5	0.1	/	/	10	23	30.3
	2	37	0.5	0.1	0.208	/	6	31	16.2
	3	40	0.5	0.1	0.128	0.803	6	34	15.0
	4	41	0.4	0.1	0.032	0.511	3	38	7.3
EOS (%) reference: 0.4-8.0	1	33	2.7	2.4	/	/	2	31	6.1
	2	37	2.6	1.9	0.832	/	2	35	5.4
	3	40	2.6	2.9	0.735	0.900	1	39	2.5
	4	41	2.2	2.0	0.334	0.509	1	40	2.4
EOSabs (109/L) reference: 0.02-0.52	1	33	0.2	0.1	/	/	2	31	6.1
	2	37	0.1	0.1	0.885	/	1	36	2.7
	3	40	0.1	0.1	0.579	0.674	1	39	2.5
	4	41	0.1	0.1	0.233	0.503	1	40	2.4
BASO (%) reference: 0-1	1	33	0.7	0.2	/	/	2	31	6.1
	2	37	0.7	0.3	0.994	/	4	33	10.8
	3	40	0.6	0.2	0.393	0.383	3	37	7.5
	4	41	0.6	0.2	0.458	0.903	4	37	9.8

BASOabs (109/L) reference: 0-0.06	1	33	0.0	0.0	/	/	0	33	0.0
	2	37	0.0	0.0	0.910	/	1	36	2.7
	3	40	0.0	0.0	0.448	0.507	1	39	2.5
	4	41	0.0	0.0	0.419	0.962	3	38	7.3
RBC (1012/L) reference: 3.8-5.1	1	33	4.5	0.7	/	/	7	26	21.2
	2	37	4.5	0.6	0.907	/	11	26	29.7
	3	40	4.5	0.6	0.614	0.692	12	28	30.0
	4	41	4.5	0.5	0.563	0.939	11	30	26.8
HCT (%) reference: 35-45	1	33	42.0	4.8	/	/	12	21	36.4
	2	37	41.8	4.3	0.795	/	12	25	32.4
	3	40	42.0	4.3	0.968	0.817	14	26	35.0
	4	41	41.8	4.1	0.830	0.854	10	31	24.4
MCV (fL) reference: 82-100	1	33	95.0	4.9	/	/	4	29	12.1
	2	37	93.8	4.4	0.292	/	1	36	2.7
	3	40	93.3	4.3	0.107	0.575	1	39	2.5
	4	41	92.6	4.4	0.027	0.527	1	40	2.4
RDW-SD (fL) reference: 38.2-49.2	1	33	46.8	6.2	/	/	10	23	30.3
	2	37	46.0	6.1	0.591	/	8	29	21.6
	3	40	45.0	6.0	0.201	0.449	8	32	20.0
	4	41	44.1	6.0	0.058	0.517	8	33	19.5
RDW-CV (%) reference: 12.1-14.3	1	33	13.5	1.2	/	/	6	27	18.2
	2	37	13.3	1.3	0.653	/	9	28	24.3
	3	40	13.1	1.2	0.205	0.403	10	30	25.0
	4	41	12.9	1.2	0.067	0.554	13	28	31.7
HGB (g/L) reference: 115-150	1	33	133.8	17.2	/	/	7	26	21.2
	2	37	135.6	16.1	0.642	/	14	23	37.8
	3	40	137.2	16.3	0.382	0.679	14	26	35.0
	4	41	136.9	16.1	0.423	0.935	13	28	31.7
MCH (pg) reference: 27-34	1	33	30.2	1.6	/	/	1	32	3.0
	2	37	30.4	1.4	0.496	/	1	36	2.7
	3	40	30.4	1.5	0.500	0.984	1	39	2.5
	4	41	30.3	1.5	0.808	0.647	2	39	4.9
MCHC (g/L) reference: 316-354	1	33	318.0	12.5	/	/	13	20	39.4
	2	37	324.3	8.7	0.014	/	8	29	21.6
	3	40	326.2	9.7	0.001	0.442	6	34	15.0
	4	41	326.8	11.0	0.000	0.295	7	34	17.1
PLT(109/L) reference: 125-350	1	33	166.3	111.2	/	/	11	22	33.3
	2	37	237.8	46.9	0.000	/	1	36	2.7
	3	40	236.5	40.5	0.000	0.930	0	40	0.0
	4	41	236.3	45.3	0.000	0.994	0	41	0.0
	1	30	0.2	0.1	/	/	8	22	26.7
	2	37	0.3	0.0	0.000	/	1	36	2.7

PCT (%) reference: 0.18-0.39	3	40	0.2	0.0	0.000	0.956	1	39	2.5
	4	41	0.3	0.0	0.000	0.929	2	39	4.9
LCR (%) reference: 17.5-42.3	1	30	30.9	8.7	/	/	4	26	13.3
	2	37	29.6	6.3	0.470	/	2	35	5.4
	3	40	29.6	7.6	0.465	0.996	4	36	10.0
	4	41	29.9	6.7	0.581	0.843	3	38	7.3
Hs-CRP (mg/L) reference: 0-0.5	1	41	0.4	0.6	/	/	6	35	14.6
	2	41	0.7	1.0	0.025	/	8	33	19.5
	3	41	0.6	0.8	0.133	0.452	8	33	19.5
	4	38	0.3	0.3	0.827	0.091	3	35	7.9
PCT (ng/mL) reference: 0-0.5	1	41	0.0	0.0	/	/	0	41	0.0
	2	41	0.0	0.0	0.934	/	0	41	0.0
	3	41	0.1	0.0	0.041	0.049	0	41	0.0
	4	39	0.1	0.1	0.026	0.834	0	39	0.0
IL-6 (pg/mL) reference: <7	1	38	11.8	25.7	/	/	7	31	18.4
	2	41	4.6	17.3	0.045	/	2	39	4.9
	3	40	2.2	1.8	0.008	0.498	3	37	7.5
	4	29	1.5	0.2	0.010	0.867	0	29	0.0
Ab (S/COI） reference:<1	1	38	364.6	330.1	/	/	0	38	0.0
	2	41	291.1	291.5	0.277	/	0	41	0.0
	3	41	278.9	281.2	0.205	0.855	0	41	0.0
	4	38	289.7	293.5	0.277	0.873	0	38	0.0
IgA (S/COI) reference:<1	1	38	4.1	2.8	/	/	2	36	5.26
	2	41	3.4	2.8	0.295	/	5	36	12.2
	3	41	3.3	2.4	0.184	0.773	4	37	9.76
	4	38	3.2	2.6	0.167	0.934	5	33	13.16
IgM (S/COI) reference:<1	1	38	3.3	7.1	/	/	22	16	57.9
	2	41	1.8	3.8	0.171	/	18	23	43.9
	3	41	1.6	4.0	0.129	0.878	14	27	34.1
	4	38	1.3	3.2	0.070	0.741	9	29	23.7
IgG (S/COI) reference:<1	1	38	18.8	5.3	/	/	0	38	0.0
	2	41	19.9	5.7	0.369	/	0	41	0.0
	3	41	20.6	5.4	0.147	0.571	0	41	0.0
	4	38	21.3	5.6	0.045	0.550	0	38	0.0
25-OH vitamin D (ng/mL) reference:5-100	1	41	28.5	9.3	/	/	0	41	0.0
	2	41	23.9	5.5	0.001	/	0	41	0.0
	3	41	24.2	4.9	0.002	0.846	0	41	0.0
	4	38	25.9	4.0	0.070	0.224	0	38	0.0
C-Peptide (ug/L) reference:0.1-10	1	41	6.5	3.1	/	/	4	37	9.8
	2	41	7.5	3.5	0.213	/	9	32	22.0
	3	41	7.9	3.3	0.095	0.667	9	32	22.0

	4	38	8.6	4.2	0.011	0.355	9	29	23.7
SAA (mg/L) reference:0-10	1	41	6.3	4.3	/	/	3	38	7.3
	2	41	5.7	3.2	0.377	/	2	39	4.9
	3	41	5.4	2.3	0.178	0.641	1	40	2.4
	4	38	5.5	2.3	0.258	0.848	1	37	2.6
FT3 (pmol/L) reference:2-4.4	1	41	3.0	0.5	/	/	1	40	2.4
	2	40	3.1	0.6	0.916	/	2	38	5.0
	3	41	3.2	1.0	0.217	0.262	4	37	9.8
	4	39	3.1	0.4	0.550	0.533	0	39	0.0
FT4 (pmol/L) reference: 12-22	1	41	17.8	2.7	/	/	5	36	12.2
	2	39	18.3	3.8	0.717	/	8	31	20.5
	3	41	20.8	11.2	0.032	0.078	7	34	17.1
	4	39	18.6	3.3	0.560	0.123	5	34	12.8
TSH (μ IU/mL) reference:0.27-4.2	1	41	2.3	2.2	/	/	3	38	7.3
	2	40	1.9	1.1	0.590	/	3	37	7.5
	3	41	3.3	4.5	0.094	0.028	7	34	17.1
	4	39	2.9	1.6	0.275	0.569	8	31	20.5
TPO (IU/mL) reference: 0-34	1	41	35.9	92.1	/	/	5	36	12.2
	2	39	15.1	11.4	0.313	/	4	35	10.3
	3	41	59.3	154.8	0.250	0.033	7	34	17.1
	4	39	18.1	21.1	0.387	0.046	4	35	10.3
TG (IU/mL) reference: 0-115	1	41	42.0	65.6	/	/	4	37	9.8
	2	40	17.3	13.3	0.086	/	0	40	0.0
	3	41	50.6	93.0	0.549	0.021	5	36	12.2
	4	39	34.2	57.9	0.589	0.258	3	36	7.7

p1, p2 were comparison between the current stage and the stage 1, the current stage and above stage, respectively. All data were analysed using the Mann–Whitney U test. P values <0.05 indicate significant differences.

Supplementary figures

Figure S1 The comparison of CT scores among 92 mild/moderate ill cases and 34 severe/critically ill cases. The average scores were compared between mild/moderate cases (red) and severe/critically cases (green). A two tailed independent sample t-test was used to show the significant difference between the two groups.

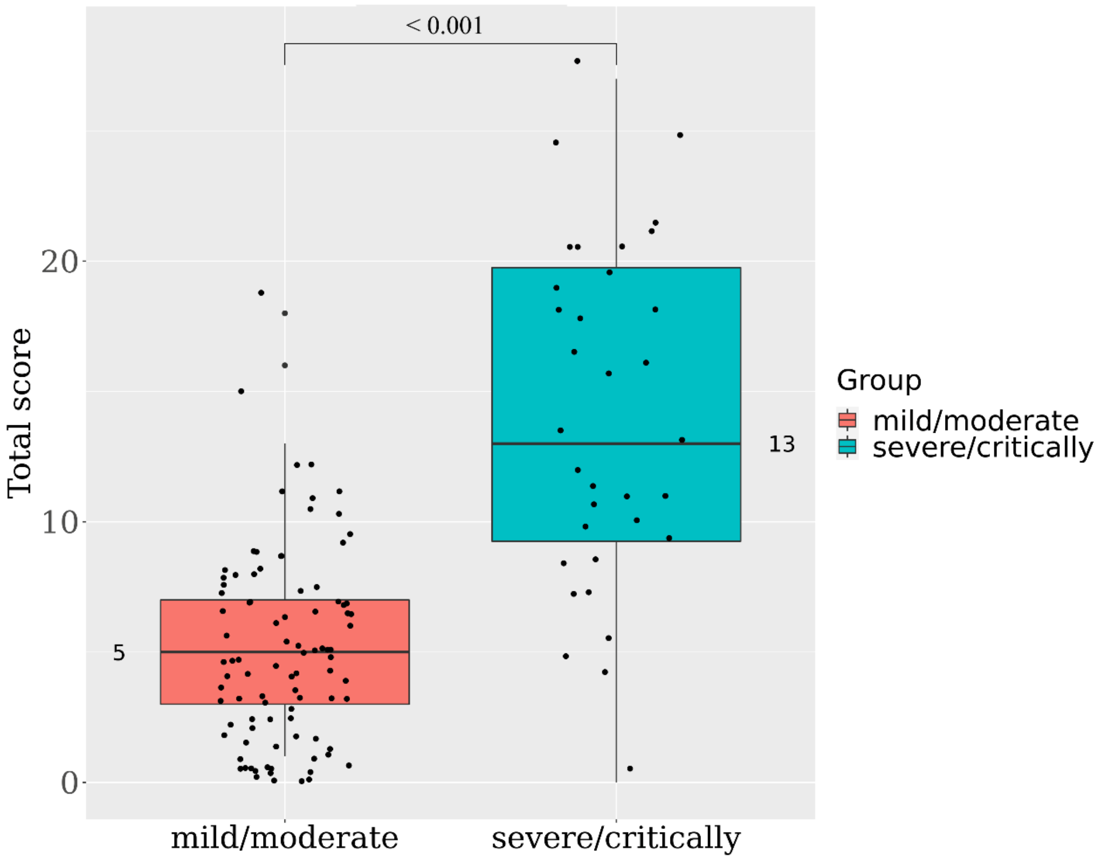

Figure S2 Proportions of patients with Ageusia and anosmia. Among imported cases, there are more patients had Ageusia/ anosmia during disease period than that in recovery. Among native cases, the contrast was the opposite.

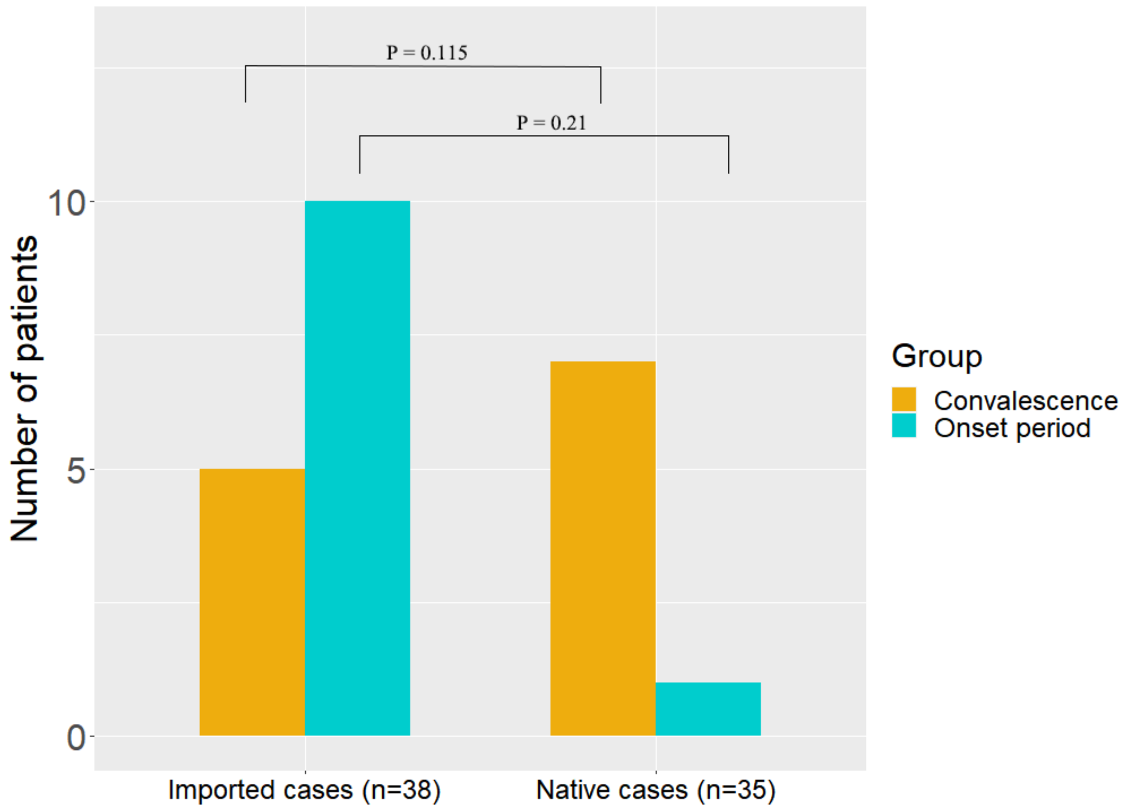

Figure S3 sampling information of the 41 re-positive patients. Each sample named as case number (age, sex/type). F, female; M, male; S, severe/critically cases; N, mild/moderate cases.

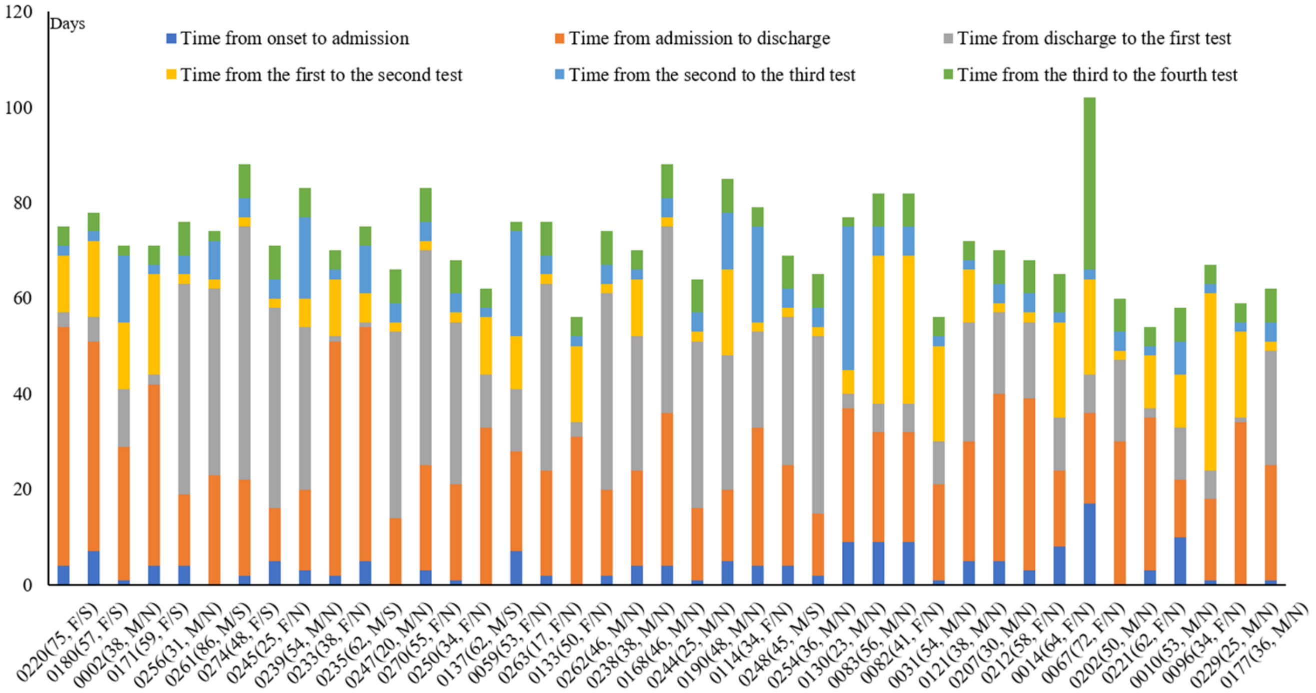

Supplement: Supplementary file 1 — Supplementary figures and tables. [file ijmsv18p0347s1.pdf]
